# Pulmonary embolism in the ICU: clinical and prognostic signification - can we predict mortality?

**DOI:** 10.1186/cc9436

**Published:** 2011-03-11

**Authors:** A Aller, M Mourelo, P Vidal

**Affiliations:** 1 University Hospital A Coruña, Spain

## Introduction

This study was to define characteristics of patients with pulmonary embolism (PE) admitted to the ICU, and to determine the usefulness of predictive models of empirical prognostic stratification.

## Methods

Retrospective study of patients who developed PE during the ICU stay or were admitted to the ICU for PE for 5 years (2005 to 2010). We analyzed: age, sex, history, diagnosis, complications and mortality. Univariate analysis using Student *t *and chi-square tests, and multivariate using logistic regression.

## Results

We found 64 patients. Mean age was 64 years (SD 16.2); 51.6% were women, 18.8% had a neoplasia, 65.5% were admitted for PE from the emergency room. The rest were: medical (18.8%), surgical (7.8%) or traumatic (6.3%). In total, 79.7% dyspnea, 34.4% chest pain, 14.1% cardiorespiratory arrest. The diagnosis was mainly by CT (71.4%), echocardiography (15.9%) and clinical (12.7%). Of patients, 92.1% had higher D-dimer, 33.3% had elevated troponin I; 66.7% had right ventricular dysfunction (RVD), 86.1% had pulmonary arterial hypertension (PAH); 57.8% metabolic acidosis; 42.2% hemodynamic instability; 44.4% catecholamines, 50% volume administration, 30% developed ARDS. Of the patients, 31.3% received systemic thrombolysis, 3.1% endovascular treatment. In 4.7% a vena cava filter was placed. In univariate analysis with regard to mortality we find significant: ARDS (*P *< 0.00), catecholamines (*P *= 0.00), acidosis (*P *= 0.01), hemodynamic instability (*P *= 0.02). In multivariate analysis: predictor of mortality SAPS II scale (*P *= 0.04, OR 0.06 (CI 0.99 to 1.12)). ROC curves for scales (Geneva, Wells, PESI), finding an area of 0.55, 0.65, 0.47, respectively. In a univariate analysis with regard to PESI (III to V), we found significant: SAPS II (*P *= 0.01), age (*P *= 0.005), PAH (*P *= 0.03), volume (*P *= 0.01), catecholamines (*P *= 0.00), hemodynamic instability (*P *= 0.00). In the multivariate analysis: SAPS II (*P *= 0.046, OR 0.071 (CI 0.86 to 0.99)). In the univariate analysis with regard to fibrinolysis: SAPS II (*P *= 0.00), PESI (*P *= 0.00), hemodynamic instability (*P *= 0.00). The median stay in ICU was 4 days, ICU mortality was 14.1%.

## Conclusions

Diagnosis of PE is primarily radiological. The majority of patients requiring ICU admission have RVD. Troponin has little sensitivity for the diagnosis of PE. Prognostic stratification scales do not seem to be reliable predictors of mortality; however, high PESI grades correlates with high severity illness. Fibrinolysis was not significantly associated with reduced mortality. Hemodynamic instability, metabolic acidosis and ARDS were independent predictors of mortality.
